# Uncovering cellular perturbations and key mediator communications in liver cancer using single-cell RNA sequencing

**DOI:** 10.3389/fimmu.2026.1868090

**Published:** 2026-07-01

**Authors:** Xuchen Ni, Jia Wang, Chao Zhang, Xianming Ge, Mengli Wang, Yan Pan, Sien Ma, Baoying Zheng, Qianqian Chen, Na Xu, Bao Zhao

**Affiliations:** 1 School of Food Science and Engineering, Joint Research Center for Food Nutrition and Health of Institute of Health and Medicine (IHM), Anhui Agricultural University, Hefei, Anhui, China; 2 Institute of Health and Medicine, Hefei Comprehensive National Science Center, Hefei, Anhui, China; 3 Department of Otolaryngology, The First Affiliated Hospital of Bengbu Medical University, Bengbu, Anhui, China; 4 Anhui Provincial Key Laboratory of Tumor Evolution and Intelligent Diagnosis and Treatment, Bengbu Medical University, Bengbu, Anhui, China; 5 Anhui Province Key Laboratory of Respiratory Tumor and Infectious Disease, First Affiliated Hospital of Bengbu Medical University, Bengbu, Anhui, China

**Keywords:** cell cycle, hepatocellular carcinoma (HCC), immunotherapy, lipid metabolism, single-cell RNA sequencing, UAP1L1

## Abstract

Liver cancer ranks as the sixth most common malignancy and the third leading cause of cancer-related death, with hepatocellular carcinoma (HCC) accounting for over 80% of cases. Its aggressive nature and late diagnosis severely limit treatment options. Tumor heterogeneity and a complex immune microenvironment further impede immunotherapy efficacy. By analyzing HCC data, we identified an epithelial subtype marked by proliferative capacity, invasiveness, and metabolic reprogramming, particularly in sugar and lipid metabolism. This subtype contributes to immune microenvironment complexity through interactions with Lipid-Associated tumor associated macrophage cells (LA_TAMs) that are linked to disease progression. A prognostic model (Tumor Epithelial Feature Signature, TEFS) revealed UAP1L1 as a computationally predicted key modulator of the cell cycle and extracellular matrix remodeling. Functional validation confirmed its clinical relevance and oncogenic role. These findings link epithelial features, metabolic dysregulation, and immune dynamics in HCC, identifying UAP1L1 as a potential target to enhance immunotherapy efficacy.

## 1 Introduction

Hepatocellular carcinoma (HCC) ranks as the sixth most common malignant tumor globally and a leading cause of cancer-related mortality (1, 2). Most HCC patients are diagnosed at advanced-stage disease at diagnosis (3), frequently with established metastasis, which leads to a poor prognosis (4, 5). Although treatments for HCC, including PD-1/PD-L1 inhibitors, have advanced considerably, the prognosis for advanced disease remains poor due to high rates of metastasis and recurrence (6–8). Consequently, identifying novel biomarkers to enhance early detection and prognostic evaluation is an urgent priority in HCC management.

Metabolic reprogramming is a hallmark of cancer development (9, 10), whereby tumor cells adapt to their microenvironment by altering their metabolism (11). These metabolic changes enable the production of onco-metabolites, fulfill biosynthetic demands, and thereby support tumor growth (12). The liver, as an immunologically privileged organ with a high tolerance to antigens from the gastrointestinal tract, also serves as a central hub for diverse metabolic and homeostatic processes (13, 14). Consequently, metabolic reprogramming is critically important in the pathogenesis of HCC (15).

Emerging evidence indicates that metabolic reprogramming is a progressive dynamic process characterized by three stages: activation, adaptation, and tolerance (16). Recent single-cell transcriptomic studies have revealed substantial metabolic heterogeneity among malignant hepatocytes and the tumor microenvironment in HCC (17). This study therefore aims to identify genes linked to metabolic reprogramming with high prognostic value in HCC. We integrated bulk RNA sequencing (bulk RNA-seq) and single-cell RNA sequencing (scRNA-seq) data from HCC samples and applied multiple analytical approaches to construct a metabolism-related prognostic gene signature. Our objective was to elucidate the relationship between these metabolic reprogramming-related genes and HCC prognosis and progression.

While numerous bulk-derived prognostic signatures have been developed for HCC (e.g., focusing on ferroptosis, necroptosis, or lncRNA networks), they primarily rely on tissue-level averages and struggle to resolve the heterogeneity of malignant epithelial states and their dynamic crosstalk with the immune microenvironment. Moreover, previous studies have often treated metabolic reprogramming, immune suppression, and myeloid remodeling as independent modules. In this study, we do not propose an entirely new framework but instead position our work as a single-cell-resolution refinement and integration of existing concepts. By integrating four public scRNA-seq datasets (265,125 cells), we identify malignant program3 (MP3), a terminal metabolic-immunotolerant epithelial program, and derive TEFS from its defining genes. This cell-state-driven approach reduces the averaging effects inherent to bulk sequencing, providing a more precise perspective on the evolution of epithelial, metabolic, and immune axes during HCC progression.

## 2 Materials and methods

### 2.1 Data collection

The TCGA-LIHC dataset served as the training cohort and was obtained from the UCSC platform. For external validation, two independent cohorts were utilized: the ICGC-LIRI-JP dataset (accessed via the ICGC Data Portal) and the OEP000321 cohort (sourced from the NODE database), comprising 158 and 212 HCC patients with associated transcriptomic and clinical data, respectively. scRNA-seq data for 39 HCC patients and 26 normal controls were retrieved from the GEO database under accession numbers GSE149614, GSE189903, GSE202642, and GSE245906. Following quality control, 265,125 high-quality single cells from this combined dataset were retained for subsequent analysis. Regarding normal controls, GSE149614 and GSE189903 provided matched adjacent non-tumor liver (NTL) tissues from the same patients. For GSE202642 and GSE245906, which lacked direct paired normal samples, available NTL tissues from these cohorts were utilized as reference controls for comparative analyses. All NTL samples were confirmed to be non-malignant based on histopathological records and absence of copy number variations (CNVs) in downstream analysis.

### 2.2 Cohort selection and clinical data processing

To ensure the robustness and reproducibility of the prognostic models, strict inclusion and exclusion criteria were applied to the clinical data from the TCGA, ICGC, and OEP000321 cohorts; specifically, only patients with a confirmed diagnosis of primary hepatocellular carcinoma (classified as “Primary Solid Tumor” to exclude metastatic or recurrent cases) and available follow-up data were included, while cases with a follow-up time of less than 30 days were excluded to minimize the impact of early post-operative mortality, and patients with missing critical clinical variables (including age, sex, race/ethnicity, and TNM staging) were excluded from multivariate Cox regression analysis to avoid bias, though these cases were retained for univariate survival analysis if overall survival data were available, with all clinical characteristics standardized prior to model construction.

### 2.3 Data processing

We processed the scRNA-seq data with the Seurat v5.1.0 R package (18). Quality control removed low-quality and potentially dying cells by filtering out cells with fewer than 200 unique molecular identifiers (UMIs) or fewer than 300 detected genes and excluding cells where mitochondrial genes constituted more than 20% of total expression. These thresholds, informed by the distribution of QC metrics and established practices, balanced data integrity with the elimination of compromised cells.

To identify and remove potential doublets, we used Doublet Finder v2.0.4. The expected doublet count was estimated at approximately 7.5–8%, in accordance with 10X Genomics guidelines, using the formula nExp_poi = round (0.08 × N × N/10000), where N represents the number of cells in the sample. Doublet prediction was performed with 20 principal components (PCs = 1:20) and the parameters pN = 0.25, pK = 0.09, nExp = nExp_poi, reuse. pANN = FALSE, and sct = FALSE. These settings followed the recommended defaults from the official Doublet Finder tutorial. All predicted doublets were subsequently excluded from the dataset before further analysis.

Data normalization, identification of highly variable genes, principal component analysis (PCA), and unsupervised clustering were performed using the standard Seurat pipeline. Prior to batch integration, PCA was computed on the top 50 highly variable genes, and the first 50 principal components (PCs) were retained. Harmony (v1.2.1) was then applied to correct technical variation across the four datasets, with sample identity specified as the batch variable. The algorithm was executed for 10 iterations. The efficacy of batch correction was quantitatively assessed using the Local Inverse Simpson’s Index (LISI). Finally, we used UMAP for dimensionality reduction and visualization.

For differential expression analysis, we identified significantly differentially expressed genes (DEGs) using the Find All Markers () function in Seurat with the Wilcoxon rank-sum test. We applied thresholds of min.pct = 0.25 and logfc.threshold = 0.25, setting only.pos = FALSE. Genes with a p-value < 0.05 were considered statistically significant.

### 2.4 Cell type identification

To define cell types, we performed differential expression analysis across clusters with the Find All Markers () function in Seurat. Marker genes were identified based on an adjusted p-value < 0.05, expression in more than 25% of cells within a cluster (min.pct = 0.25), and an absolute log2-fold change > 0.25. The top differentially expressed genes for each cluster were designated as cluster-specific highly expressed genes. We then compared these specific markers against curated reference databases, Cell Marker and PanglaoDB, to infer the most probable cell type for each cluster. Manual annotation was subsequently performed by evaluating typical lineage markers and established expression patterns of cell-type-specific genes. To support and cross-validate these manual annotations, we also utilized the cell marker accordion package, which automatically annotates cell populations by integrating positive and negative markers from its internal database. The results from cell marker accordion served as a secondary reference and were aligned with our primary marker-based annotation. Final cell type labels were assigned by integrating evidence from marker gene analysis, database matching, and cell marker accordion predictions.

### 2.5 CNV analysis

Copy number variations (CNVs) from single-cell RNA sequencing data were inferred using CopyKAT (v1.0.7) (19). CopyKAT predicts large-scale chromosomal CNVs based on normalized gene expression profiles and genome segmentation, distinguishing tumor cells from normal cells. Default parameters were applied to generate a CNV matrix for each cell, which was subsequently used for tumor cell identification and subpopulation analysis.

### 2.6 Cell communication analysis

The Cell Chat v2.1.2 R package (20) was used to infer cell-cell communication networks within the tumor microenvironment from receptor-ligand interactions. Communication probability and interaction counts were calculated to construct these networks. The interactions between any two cell populations were visualized, and scatter plots displayed the major signaling sources and targets in a two-dimensional space. This approach identified the principal contributors of outgoing or incoming signals, particularly among immune cell types. A pattern recognition method was then employed to determine how multiple immune cell types and signaling pathways coordinate. Note that CellChat infers communication probabilities based on ligand-receptor expression co-occurrence and should be interpreted as hypothesis-generating rather than definitive mechanistic evidence.

### 2.7 Trajectory analysis

To estimate the relative differentiation potential of malignant epithelial cell states, we applied CytoTRACE2 (21), a deep learning-based method that predicts cellular potency from single-cell RNA-sequencing data. Pseudotime trajectories were subsequently constructed using the Monocle2 (22) algorithm, which reduces high-dimensional gene expression data into a low-dimensional space and orders cells along branching trajectories. Dynamic expression patterns were then visualized with the ‘plot_pseudotime_heatmap’ function.

### 2.8 Prognostic modeling using MIME-integrated machine learning algorithms

To construct a robust prognostic model, we employed MIME (23), a flexible machine learning framework designed for the analysis of high-dimensional omics data. This framework integrated ten algorithms, such as Lasso, Elastic Net, Random Forest, CoxBoost, and SuperPC, to perform both modeling and feature selection in survival analysis.

Transcriptomic features (mRNA only) significantly associated with overall survival in univariate Cox regression (P < 0.01) served as the input feature matrix. Prior to model construction, gene expression data from all training cohorts were normalized via Z-score standardization. The MIME preprocessing module ensured uniform scaling and removed batch effects arising from different platforms.

To construct a robust prognostic model, we employed the MIME framework, which integrates penalized regression and ensemble learning algorithms. Prior to modeling, candidate genes were pre-screened via univariate Cox regression (P < 0.01), reducing the high-dimensional transcriptomic profile to 10 prognostic candidates. The final signature was then derived using the StepCox (forward-backward) + Random Survival Forest (RSF) algorithm. RSF, an ensemble learning approach leveraging bootstrap resampling and random feature selection, inherently mitigates overfitting by reducing variance and enhancing generalizability. Model construction was performed in the training cohort (TCGA-LIHC, n = 368), yielding a parsimonious five-gene signature. This low dimensionality (EPV > 20) minimizes the risk of over-parameterization. Crucially, the final TEFS model was selected based on predictive stability across two independent external validation cohorts (ICGC, n = 158; OEP000321, n = 212) rather than solely on training-set metrics, aligning with the MIME framework’s design to prioritize generalizability over peak training performance.

The TEFS model’s interpretability was assessed using feature importance scores, Kaplan-Meier risk stratification, and time-dependent ROC curves. These visualizations elucidated the individual prognostic contribution of each model gene.

### 2.9 Immune infiltration evaluation

The CIBERSORT (24) algorithm quantified immune cell infiltration levels in HCC patients and explored differences in immune cell abundance between high- and low-risk patient groups. Pearson correlation analysis assessed the relationship between immune cell abundance and risk scores. To investigate potential differences in immune function, we employed single-sample gene set enrichment analysis (ssGSEA) to obtain enrichment scores. The Wilcoxon test was then used to compare immune function between the high- and low-risk groups.

### 2.10 Kaplan–Meier plotter database analysis

The predictive value of TEFS in hepatocellular carcinoma was analyzed using the survminer software package. Patients were stratified into high- and low-expression groups based on their TEFS levels. Overall survival (OS) was then compared between these two cohorts.

### 2.11 Cell culture and transfection

The human hepatocellular carcinoma cell lines Huh7, HepG2, and mouse Hepa1-6 (Hycyte Bioscience Co., Ltd.), were cultured in DMEM (KGM12800-500, KeyGEN Bio TECH) supplemented with 10% fetal bovine serum (FBS) (A5256701, Gibco) at 37 °C in a 5% CO_2_ atmosphere. *UAP1L1* knockout Huh7, HepG2, or *Uap1l1* knockout Hepa1–6 cells were generated using the CRISPR-Cas9 knockout system. Briefly, the lenti-CRISPR V2 vector expressing either *UAP1L1* gRNA (CCTTGGGGTAGGTCACGCCC) or *Uap1l1* gRNA (GTGACCAAGAGACACGCCTG) was packaged in 293T cells along with psPAX2 and pMD2G. After infection with 0.2 μm filtered supernatants, cells were selected with puromycin (2 μg/mL for 3 days).

### 2.12 CCK-8 assay

Cells were seeded in 96-well plates containing DMEM supplemented with 10% FBS. After adding 10 μL of CCK-8 reagent to each well, the plates were incubated at 37 °C for 2–4 hours. Absorbance at 450 nm was measured with a microplate reader at 0, 24, 48, 72, and 96 hours. Each experiment was performed in triplicate.

### 2.13 Wound healing assay

To assess the impact of UAP1L1 on HCC cell migration, we conducted scratch-wound healing assays. Cells were seeded in 6-well plates at a density of 3 × 10^6^ cells per well and cultured at 37 °C with 5% CO_2_ until they formed a confluent monolayer. A uniform scratch was then introduced in each well using a pipette tip, after which the wells were gently washed with phosphate-buffered saline (PBS). We monitored and imaged wound closure at 0, 24, 48, and 72 hours following the scratch. The resulting scratch areas were quantified with ImageJ software. Each experiment was performed in triplicate to confirm reproducibility.

### 2.14 Transwell migration assay

A Transwell migration assay was conducted using a 24-well plate with 8 μm pores (Corning Costar). Cells were suspended in serum-free medium at 5 × 10^4^ cells/mL, and 100 μL of this suspension was placed in the upper chamber. The lower chamber contained 600 μL of complete medium supplemented with 10% FBS. Following a 48-hour incubation, cells were fixed with 4% paraformaldehyde for 30 minutes and then stained with 0.1% crystal violet for 15 minutes at room temperature. Non-migratory cells on the upper membrane surface were removed with a cotton swab, and migrated cells were counted in three random microscopic fields (Olympus Corporation) at 20× magnification. Each experiment was performed in triplicate.

## 3 Results

### 3.1 Single-cell transcriptomic analysis reveals the cellular composition and functional remodeling in the HCC microenvironment

**Figure 1:**
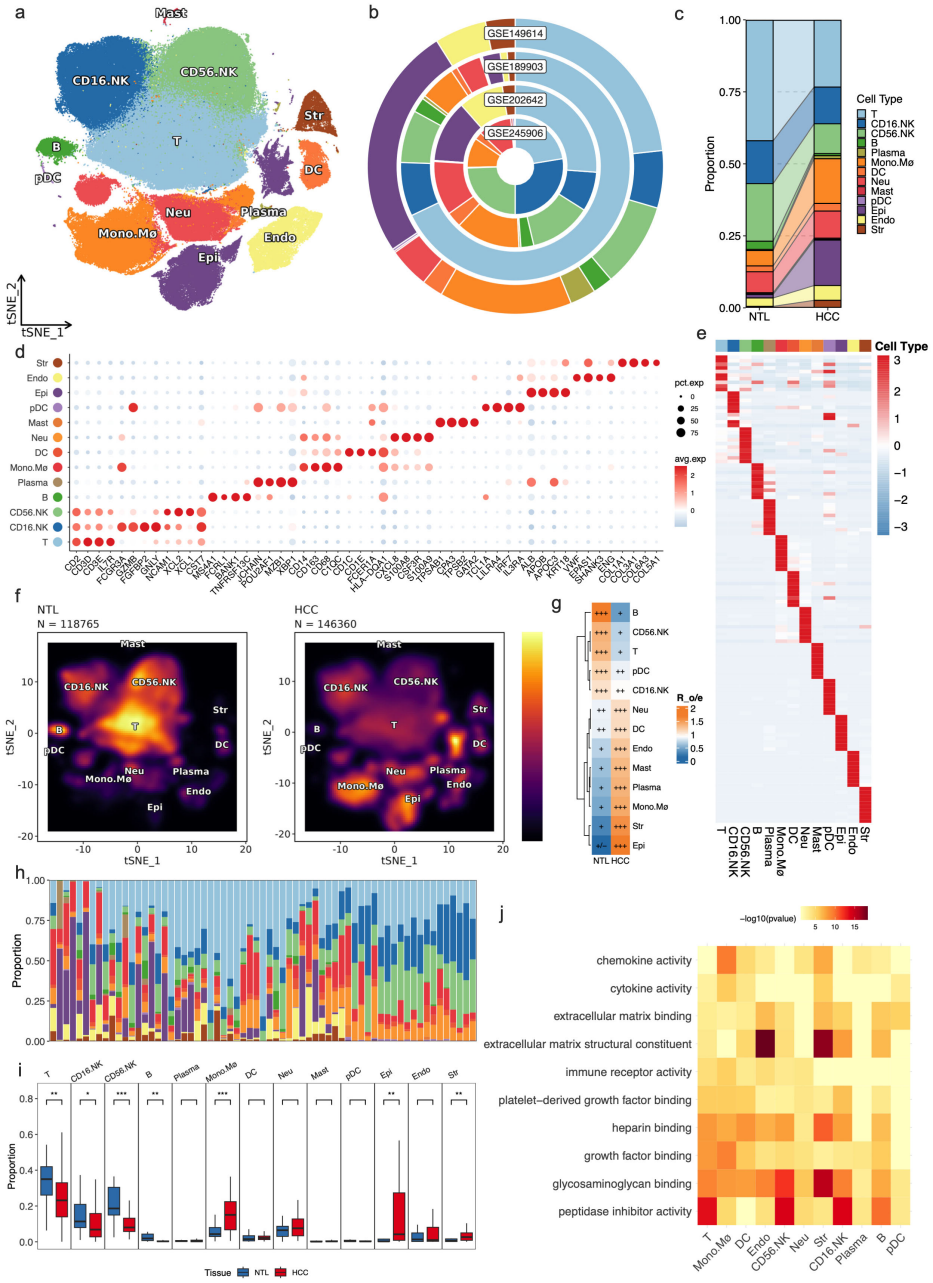
Single-cell transcriptomic analysis reveals cell type composition and functional remodeling in hepatocellular carcinoma. **(A)** t-SNE dimensionality reduction showing the integrated cell distribution map. Thirteen major cell types were identified, each represented by a distinct color. **(B)** Pie chart showing the proportion of different cell types across datasets from different sources. **(C)** Bar chart showing the proportion of different cell types in tumor and normal groups. **(D)** Expression profiles of characteristic marker genes across different cell types. **(E)** Heatmap displaying the top 10 highly expressed genes specific to each cell type, illustrating the transcriptional characteristics of different cell types. **(F)** Density distribution plot of each cell type in tumor and normal groups, showing differences in cell abundance between groups. **(G)** Tissue preference of each cluster estimated by the STARTRAC-dist index: +++, Ro/e > 1; ++, 0.8 < Ro/e ≤ 1; +, 0.2 < Ro/e ≤ 0.8; +/-, 0 < Ro/e ≤ 0.2; -, Ro/e = 0. **(H)** Stacked bar plot showing the proportional distribution of different cell types across samples. **(I)** Composition ratio of tumor and normal groups within each cell type. **(J)** Representative GO functional pathways enriched in upregulated DEGs in each cell type. *, P<0.05; **, P<0.01; ***, P<0.001.

Data from four public datasets (GSE149614, GSE189903, GSE202642, GSE245906), comprising 65 samples, including 26 normal liver tissues and 39 HCC tumor, were integrated for this study (**Figures 1a–c**; **Supplementary Table 1**). To rigorously evaluate the success of batch integration, we quantified inter-batch mixing using the Local Inverse Simpson’s Index (LISI). LISI scores significantly increased following Harmony correction, confirming effective removal of technical variation while preserving biological heterogeneity (**Supplementary Figures 1a–c**). Following stringent quality control and the removal of suspected doublets, 265,125 high-quality single cells were retained for analysis. Using established cell-specific marker genes, we identified 13 distinct cell types: including T cells (expressing CD3D, CD3E), CD16^+^ NK cells (FCGR3A, GZMB), CD56^+^ NK cells (low FCGR3A, high NCAM1), B cells (MS4A1, FCRL1), plasma cells (JCHAIN, POU2AF1), monocytes and macrophages (CD14, CD163), dendritic cells (CD1C, FCER1A), neutrophils (FCGR3A, S100A8), mast cells (TPSAB1, CPA3), plasmacytoid dendritic cells (TCL1A, LIRA4), epithelial cells (ALB, APOB), endothelial cells (VWF, EPAS1), and stromal cells (COL1A1, COL3A1) (**Figure 1d**). A transcriptional expression heatmap confirmed these distinct cell types, clearly delineating their unique gene signatures (**Figure 1e**).

To systematically assess the impact of HCC on cellular composition, we first compared the abundance of each cell type between tumor and normal tissues. Density map visualizations revealed distinct spatial distribution patterns, such as the predominant aggregation of lymphocytes in normal tissue and the preferential accumulation of myeloid and epithelial cells in tumor samples (**Figure 1f**). Consistent with this, epithelial cells, monocytes and macrophages were significantly enriched in the tumor group (**Figure 1c**). Analysis using the tissue distribution deviation from random expectation (R(o/e)) (25) confirmed these findings, indicating significant clustering of epithelial cells, stromal cells, and monocytes and macrophages in tumors, whereas NK cells, T cells, and B cells were more abundant in normal tissues (**Figure 1g**). Cell proportion statistics further demonstrated notable inter-sample heterogeneity, with increased proportions of epithelial cells, stromal cells, and monocytes and macrophages in the tumor group, alongside decreased proportions of B cells, T cells, and NK cells, reflecting the remodeling of immune microenvironment in HCC (**Figures 1h, i**).

To elucidate functional changes in key cell types during HCC progression, we conducted Gene Ontology (GO) enrichment analysis on differentially expressed genes from each cell type within the tumor group (**Figure 1j**). Monocytes and macrophages exhibited significantly enrichment for chemokine activity and growth factor binding pathways, consistent with the activation of their immunoregulatory and tumor-promoting functions (26). Endothelial cells and stromal cells showed upregulation in pathways related to the extracellular matrix composition, indicating their potential roles in remodeling the tumor microenvironment and supporting tumor development (27).

### 3.2 The cellular and functional synergistic reshaping in the HCC tumor microenvironment

**Figure 2:**
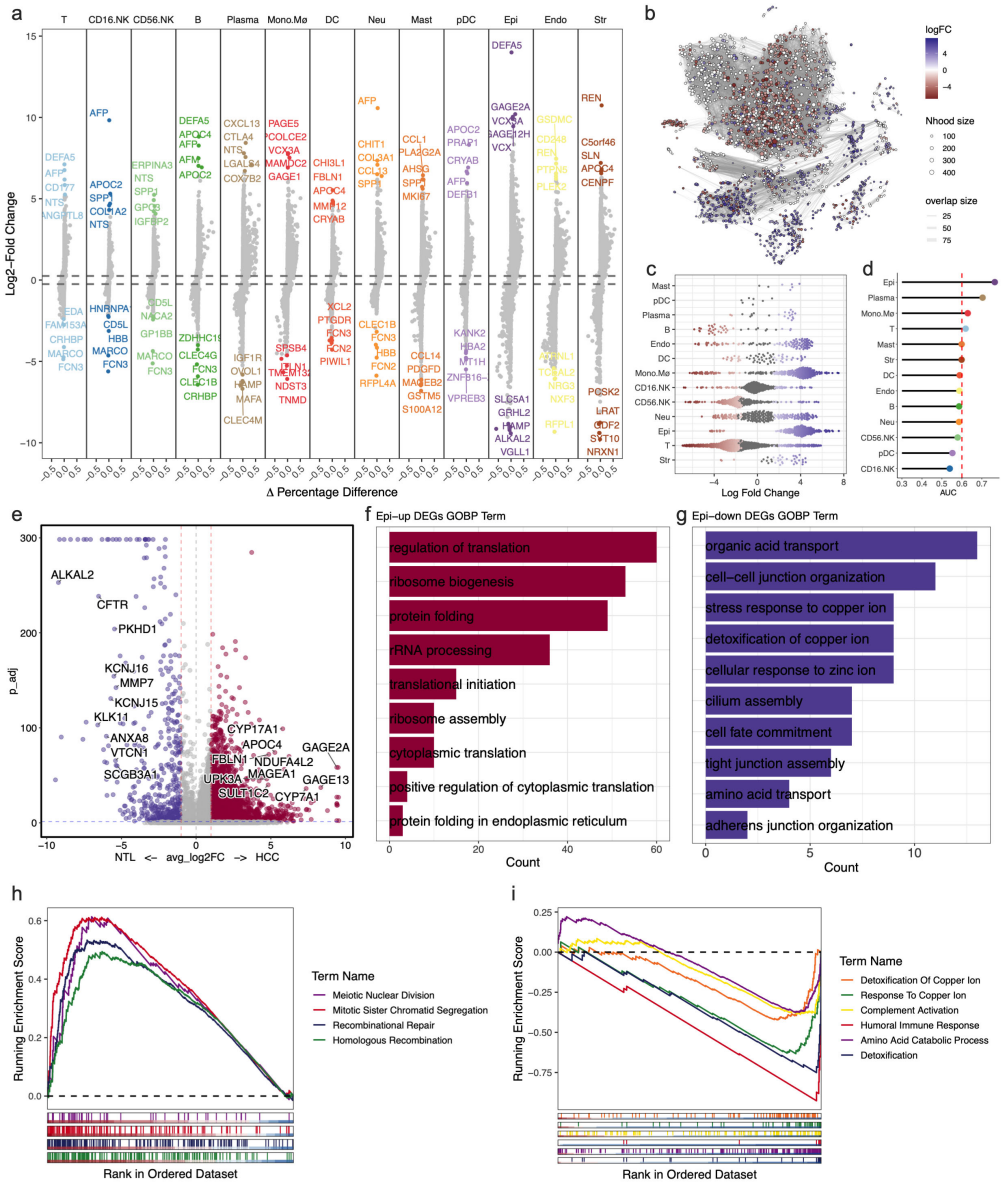
Multi-dimensional analysis reveals transcriptomic remodeling and functional changes in cell types in HCC. **(A)** Differentially expressed genes (DEGs) between tumor and normal groups across cell types, were analyzed using the Wilcoxon rank-sum test. **(B)** Differential abundance analysis using the Milo k-nearest neighbor (kNN) algorithm. **(C)** Statistical results of the Milo kNN differential abundance analysis. **(D)** Augur analysis framework assessing the degree of transcriptomic perturbation in each cell subtype between biological states. **(E)** Volcano plot of DEGs in epithelial cells between tumor and normal groups. **(F)** GO functional enrichment analysis of DEGs in tumor epithelial cells. **(G)** GO functional enrichment analysis of DEGs in normal epithelial cells. **(H)** GSEA pathway enrichment analysis of DEGs in tumor epithelial cells, showing representative signaling pathways. **(I)** GSEA pathway enrichment analysis of DEGs in normal epithelial cells.

We systematically analyzed transcriptional characteristics across cell types in HCC and adjacent normal liver tissues. Differential expression analysis revealed robust molecular alterations (**Figure 2a**): the classic HCC tumor markers AFP and GPC3 were broadly upregulated not only in epithelial tumor cells (28) but also in immune and stromal populations, indicating a global ‘tumorization’ of the microenvironment. Meanwhile, APOC2/4, AKR1B10 and SPP1 were consistently elevated across nearly all cell subsets, highlighting lipid metabolic reprogramming and extracellular matrix remodeling as core features of HCC (29–31). In contrast, CLEC1B, LYVE1, MARCO and HAMP were uniformly downregulated, reflecting a significant loss of normal hepatic scavenger function and iron homeostasis (32, 33). Notably, FOXP3, CCR8 and CD274 (PD-L1) were significantly induced in T cells and dendritic cells, while cytotoxic molecules such as GZMK were markedly reduced in NK cells, demonstrating a transition of the immune microenvironment toward a protumorigenic, immunosuppressive state, where PD-L1 acts as a key target that is widely expressed in various cancers, including HCC-related epithelial cells, and mediates immune escape (34).

Following the characterization of molecular changes, we examined disease-related alterations in cell composition and transcriptional status. Differential abundance analysis showed that the proportions of epithelial cells, endothelial cells, and monocytes/macrophages were significantly increased in tumors, indicating parenchymal expansion, active angiogenesis, and altered immune infiltration patterns (**Figures 2b, c**). Concurrently, an assessment of transcriptional perturbation levels revealed significant gene expression remodeling in epithelial cells, plasma cells, monocytes/macrophages, and T cells in HCC, suggesting these cell types exhibit high biological activity during disease progression (**Figure 2d**).

Given the prominent proportion and notable transcriptional changes of epithelial cells, we performed an in-depth functional enrichment analysis on this population. Gene Ontology analysis showed that upregulated genes were primarily enriched in processes such as the cell cycle, chromosome segregation, and the DNA damage response, indicating tumor cells activate core proliferation programs and respond to replication stress to sustain growth (35). Downregulated genes were concentrated in metabolic and immune regulatory functions, reflecting a loss of normal differentiation and homeostatic capacity (**Figures 2e–g**). These findings corroborated and extended the GSEA results: GSEA not only detected activation of genomic stability pathways like homologous recombination but also specifically highlighted the suppression of key pathways such as the humoral immune response (**Figures 2h, i**). Collectively, malignant progression in HCC is driven by tumor cell-autonomous proliferation and adaptation mechanisms, complemented by systemic immunosuppression in the tumor microenvironment.

### 3.3 Dynamic changes of the epithelium in HCC

**Figure 3:**
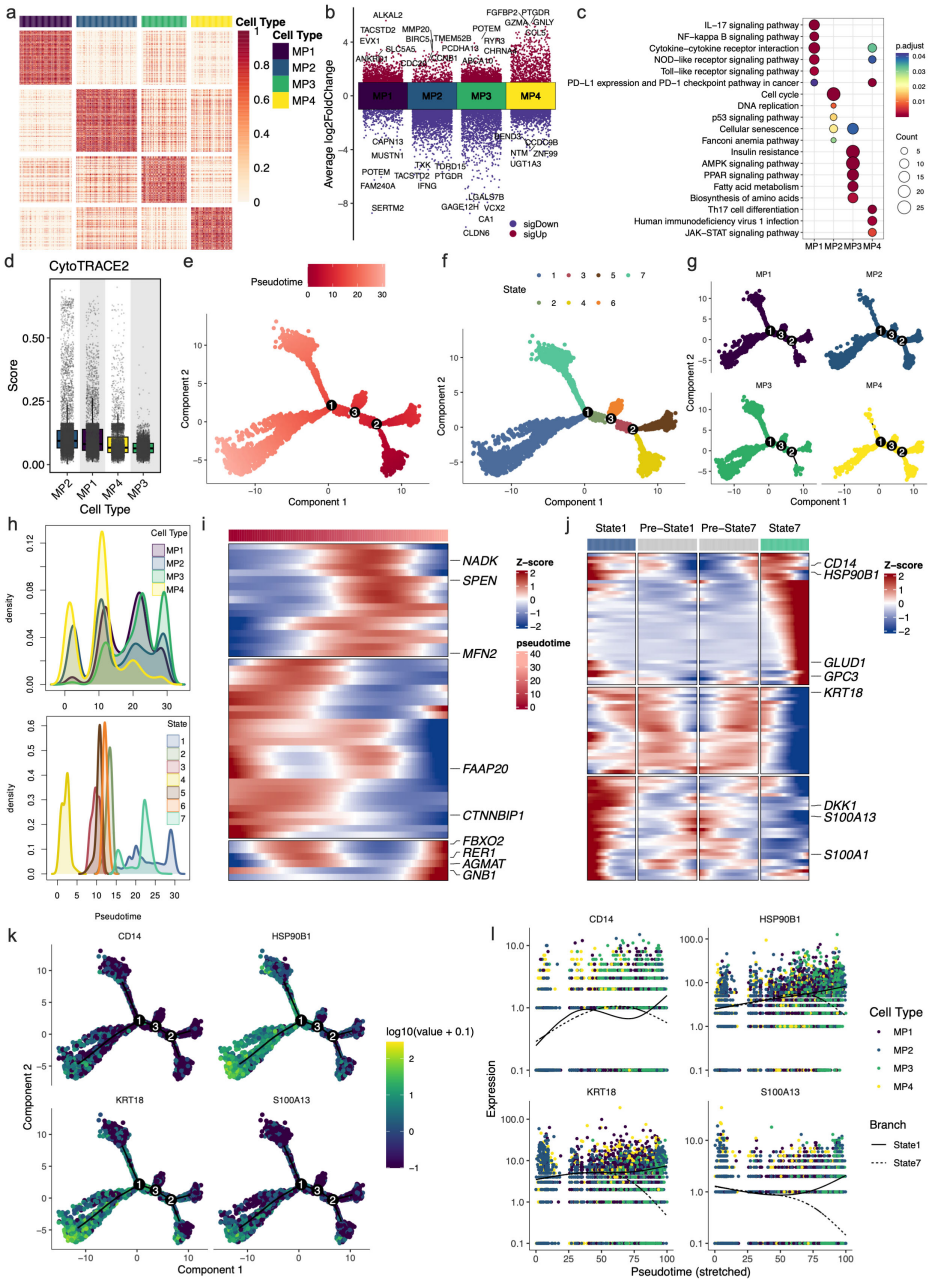
Pseudotime analysis reveals the dynamic transition trajectory of HCC malignant epithelial cells. **(A)** GeneNMF identified 4 programs from malignant cells. **(B)** DEGs between different programs were analyzed using the Wilcoxon rank-sum test. **(C)** GO functional enrichment analysis of DEGs between programs, highlighting representative signaling pathways. **(D)** CytoTRACE analysis of differentiation states among different programs. **(E)** Developmental pseudotime analysis inferred from Monocle. **(F)** Cell-state distribution inferred from Monocle analysis. **(G)** Distribution characteristics of different programs and cell states along pseudotime. **(H)** Distribution of different programs as inferred by Monocle analysis. **(I)** Dynamic transcriptional features of key genes along pseudotime. **(J)** BEAM analysis identifying key transition node DEGs. **(K)** Expression distribution of selected genes along pseudotime. **(L)** Expression distribution of selected genes along pseudotime in different branches.

Considering that epithelial cells are the primary source of malignancy in HCC, we analyzed these cells in depth. To isolate malignant cells from the epithelial cell population, we utilized the CopyKAT tool to analyze the epithelial cells (comprising a sample size of over 100 cells, using T and NK cells as references). The results demonstrated a significant separation between the identified malignant cells and the non-malignant cells. (**Supplementary Figure 2**). Using non-negative matrix factorization (NMF), we identified four distinct transcriptional states among the malignant cells (**Figure 3a**). These states exhibited entirely different sets of differentially expressed genes (DEGs) (**Figure 3b**). GO enrichment analysis of the state-specific DEGs revealed distinct functional profiles (**Figure 3c**): MP1 showed upregulation in IL-17, NF-κB, TLR, and NOD-like receptor signaling, as well as PD-L1 expression, suggesting a pro-inflammatory tumor microenvironment (36). MP2 was enriched for Cell cycle, DNA replication, p53, and Fanconi anemia pathways, reflecting canonical proliferation signals. The co-occurrence of p53 and Fanconi anemia pathways implies genomic instability and a heightened DNA damage response, features common in aggressive tumors (37, 38). MP3 demonstrated upregulation in Insulin resistance, AMPK, PPAR, and fatty acid metabolism pathways, corresponding to metabolic reprogramming and dysregulation of sugar and lipid metabolism consistent with the Warburg effect (39). MP4 was enriched for Th17 cell differentiation, PD-L1, HIV-1 infection, and JAK-STAT signaling, pathways linked to adaptive immunity. The association of Th17 differentiation with PD-L1 points to an immune-inhibitory microenvironment (40). These functional modules correspond to different facets of tumor biology, providing insight into liver cancer progression. Subsequent CytoTRACE2 analysis positioned MP3 at the endpoint of a developmental trajectory, delineating a progression from “Proliferation” to “Inflammation”, then “Immune Evasion”, and finally “Metabolic Tolerance”, which implies a functional evolution within the tumor microenvironment at single-cell resolution (**Figure 3d**).

To characterize the dynamic transitions among malignant epithelial cell states, we ordered these cells along a pseudotemporal trajectory using Monocle2. The analysis revealed that MP3 was primarily enriched at the terminal pseudotime state, whereas MP2 and MP4 were more prevalent at earlier stages, reinforcing that proliferative activity was highest during initial tumor progression (**Figures 3e–h**). An immunosuppressive microenvironment, arising from sustained immune activation, was associated with the middle phase and coincided with distinct metabolic reprogramming.

Along this trajectory, high expression of FAAP20 and CTNNBIP1 during the early stage supported rapid proliferation and genomic maintenance in hepatocellular carcinoma cells (41, 42). Mid-trajectory upregulation of NADK, SPEN, and MFN2 facilitated adaptation to inflammatory stress and immune evasion (43). Terminal-stage elevation of FBXO2, RER1, AGMAT, and GNB1 promoted metabolic adaptation and microenvironmental tolerance (44–47). This stage-specific gene expression validates the inferred trajectory (**Figure 3i**). At the terminal branch point, tumor cell fate diverged into two distinct subgroups: one displayed robust immune-regulatory features and a preference for amino acid metabolism, marked by high expression of GPC3, CD14, and GLUD1, suggesting interplay with tumor-associated macrophages and glutamine-driven metabolic reprogramming (48–50). The other branch exhibited suppressed proliferative signals, such as DKK1, alongside activated stress responses, including S100 proteins, indicating a slow-cycling or stress-tolerant phenotype (**Figures 3j–l**).

### 3.4 Heterogeneity and functional remodeling of immune cells in the microenvironment of HCC

Immune cells are a key component of the tumor microenvironment and are closely associated with tumor progression (51). Given our prior observation of significant dysregulation in the immune response and tumor microenvironment within the tumor group, we conducted a detailed analysis of T cell, monocyte, and macrophage subsets in the dataset.

**Figure 4:**
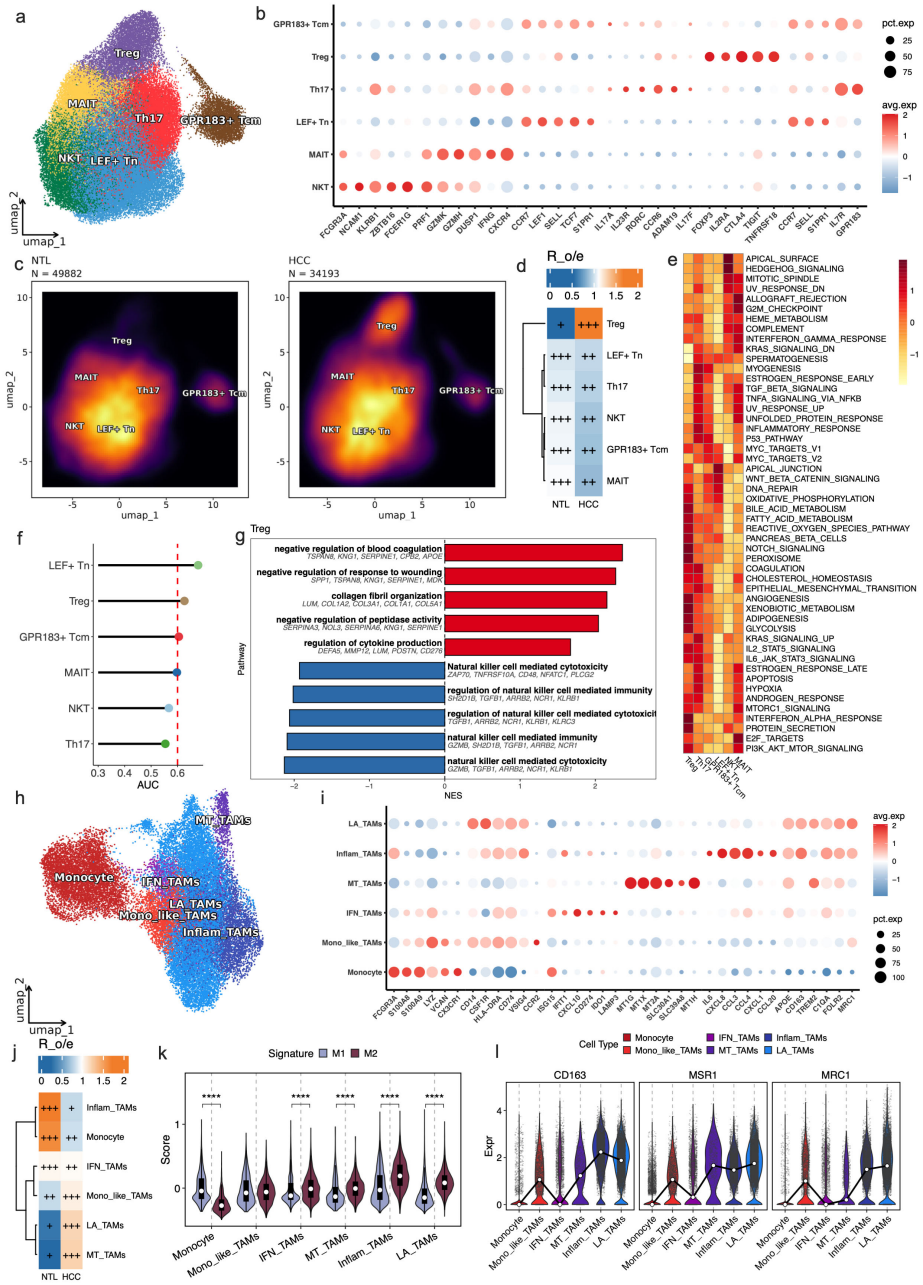
Single-cell transcriptomic analysis reveals the heterogeneity of lymphoid and myeloid cells in HCC. **(A)** UMAP dimensionality reduction showing the integrated cell distribution map. Six distinct T cell subsets were identified, each represented by a distinct color. **(B)** Dot plot showing the marker genes of six T cell subsets. **(C)** Grouped density plot showing the intergroup distribution of T cell subsets. **(D)** Tissue preference of each cluster estimated by the STARTRAC-dist index: +++, Ro/e > 1; ++, 0.8 < Ro/e ≤ 1; +, 0.2 < Ro/e ≤ 0.8; +/-, 0 < Ro/e ≤ 0.2; -, Ro/e = 0. **(E)** Heatmap of HALLMARK pathway enrichment in T cell subsets. **(F)** Augur analysis framework assessing the degree of transcriptomic perturbation in T cell subsets between biological states. **(G)** Bar plot of GSEA enrichment analysis for the Treg subset. **(H)** UMAP dimensionality reduction showing the integrated cell distribution map. Six distinct myeloid cell subsets were identified, each represented by a distinct color. **(I)** Dot plot showing the marker genes of six myeloid cell subsets. **(J)** Tissue preference of each cluster estimated by the STARTRAC-dist index: +++, Ro/e > 1; ++, 0.8 < Ro/e ≤ 1; +, 0.2 < Ro/e ≤ 0.8; +/-, 0 < Ro/e ≤ 0.2; -, Ro/e = 0. **(K)** Violin plot of M1/M2 scores across six myeloid cell subsets. **(L)** Violin plot of differentially expressed genes in myeloid cell subsets.****, P<0.0001.

Using unsupervised clustering, we identified six lymphocyte subtypes, which we annotated according to their highly expressed marker genes to confirm their identities (**Figures 4a, b**). The overall distribution of these lymphocyte subtypes differed between the tumor and normal groups. Notably, regulatory T (Treg) cell abundance was significantly higher in the tumor group, whereas other effector cell subsets showed a slight decrease, indicating enhanced immunosuppression and tumor-driven immune remodeling within the microenvironment (**Figures 4c, d**).

To characterize the functional states of distinct T-cell subgroups, we assessed pathway activation across subtypes using MSigDB hallmark gene sets. The analysis revealed distinct patterns of pathway activity. Treg cells, for instance, exhibited significant activation of the INTERFERON_GAMMA_RESPONSE and PEROXISOME pathways, underscoring their dual roles in immunoregulation and metabolic control; their high-energy demands were further indicated by activation of the GLYCOLYSIS, BILE_ACID_METABOLISM, and CHOLESTEROL_HOMEOSTASIS pathways. Among cell cycle and proliferation-related pathways, GPR183^+^ central memory T (Tcm) cells showed marked activation of the MITOTIC_SPINDLE pathway, while MYC_TARGETS_V1 was highly active in natural killer T (NKT) cells (**Figure 4e**). Analysis of transcriptional differences between tumor and normal groups using the Augur framework indicated that LEF^+^ naive T (Tn) subtypes underwent the most pronounced changes, followed by Treg cell subsets (**Figure 4f**).

GSEA analysis of Treg cells in HCC and NTL tissues revealed a pro-tumorigenic functional shift within the tumor microenvironment (**Figure 4g**). In HCC, Treg cells exhibited significant activation of pathways related to tissue remodeling, immune suppression, and tumor progression, such as the negative regulation of blood coagulation, negative regulation of the response to wounding-where SPP1 was a core gene-collagen fibril organization, negative regulation of peptidase activity, and regulation of cytokine production. These activated pathways collectively foster extracellular matrix deposition, tumor invasion, and immune evasion. Conversely, pathways mediating anti-tumor immune surveillance were suppressed in HCC, as robust enrichment for NK cell-mediated cytotoxicity and immunity pathways was observed exclusively in NTL tissues. This suppression of NK cell function underscores the immune-suppressive role of Treg cells in HCC, which facilitates tumor progression and immune escape.

In the mononuclear and macrophage analyses, we identified six distinct cell subgroups and characterized their functional marker expression (**Figures 4h, i**). Cell abundance analysis revealed that, with the exception of inflammatory Tumor-Associated Macrophages (Inflam_TAMs), all other TAM populations were significantly upregulated in the tumor group (**Figure 4j**). We further assessed these subgroups using M1 and M2 polarization scores. Monocyte cells exhibited higher M1 than M2 scores, whereas most other subgroups—except for Mono-like_TAMs-displayed an M2-polarized state (**Figure 4k**). This polarization profile suggests a potential pro-tumor role for these populations. Although Inflam_TAMs were predominantly detected in normal tissues, their distinct M2-like polarization signature suggests a potential pre-existing immunosuppressive state that may be co-opted during early tumorigenesis, whereas LA_TAMs represent the tumor-enriched terminal state driving established immunosuppression.

M2-polarized macrophages upregulated CD163, IL1B, IRF1, and MSR1, further clarifying their tumor-promoting mechanisms (**Figure 4l**). Compared to CD163^dim^ macrophages, CD163^hi^ macrophages demonstrated enhanced involvement in blood vessel development, extracellular matrix remodeling, and wound healing (52). CD163-expressing macrophages can also secrete IL-10 and TGF-β, cytokines known to inhibit T-cell antitumor activity (53). IL1B activation drives tumor proliferation and invasion via the NF-κB pathway, induces VEGF production to promote angiogenesis (54), and recruits MDSCs to intensify immunosuppression (55). IRF1 promotes PDL1 expression in tumor and myeloid cells, facilitating immune escape (56, 57). Furthermore, MSR1 expression allows TAMs to uptake lipids from the tumor microenvironment, forming lipid-laden foam cells with heightened immunosuppressive and pro-angiogenic phenotypes (58, 59).

### 3.5 The activation of multiple types of immune signaling in tumors

**Figure 5:**
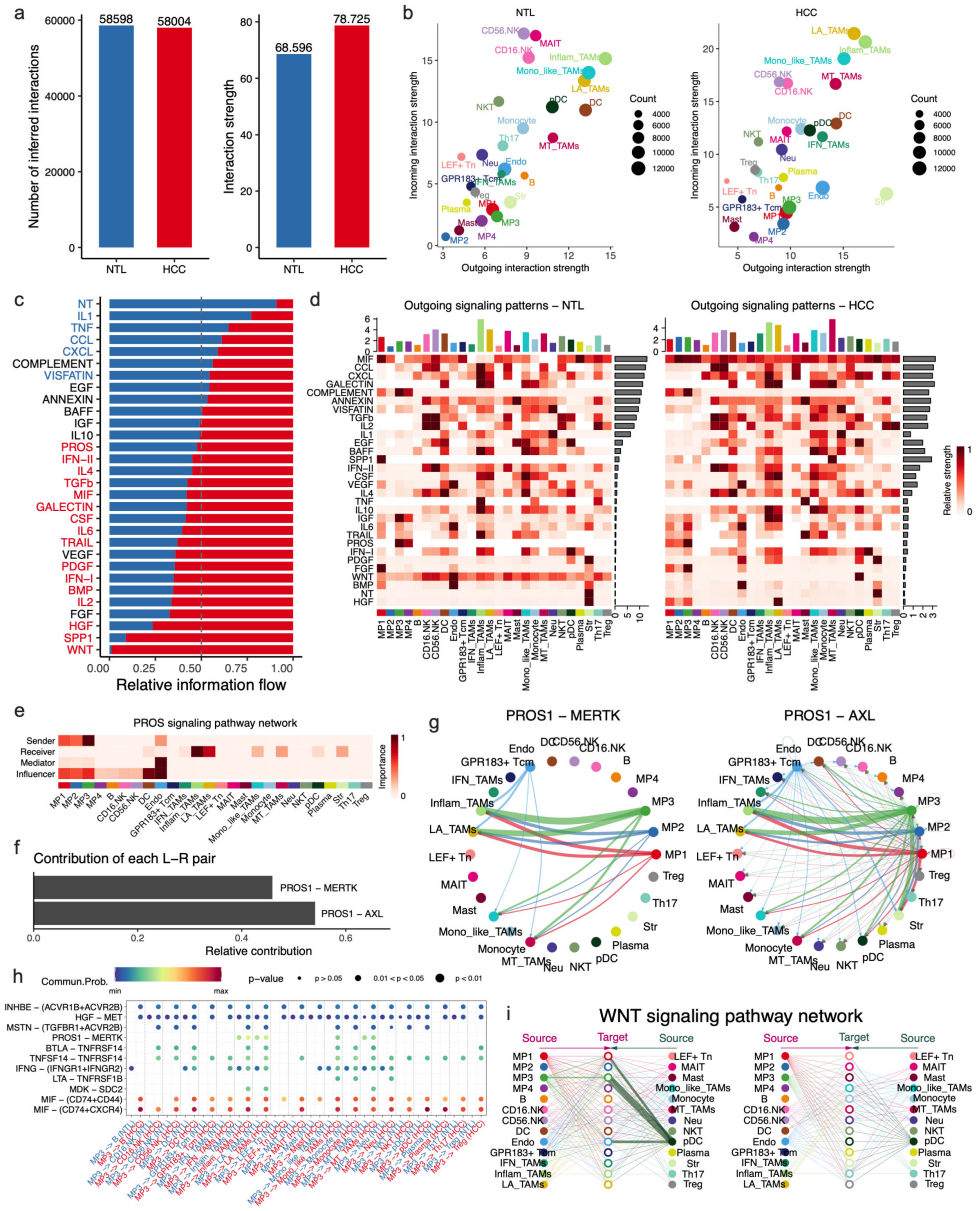
Cell communication network analysis reveals signal transduction remodeling in HCC microenvironment. **(A)** Quantitative comparison of cell communication networks between tumor and normal groups, including the analysis of the number of signaling pathways and signal strength. **(B)** Scatter plot showing the incoming and outgoing signal strength of cell subtypes in tumor and normal groups, illustrating the roles of each subtype in the signaling network. **(C)** Comparative analysis of relative information flow for each signaling pathway between tumor and normal groups. **(D)** Heatmap displaying the outgoing signal strength of different cell types in tumor and normal groups. **(E)** Network localization and functional identification of cell types involved in the PROS signaling pathway. **(F)** Contribution analysis of key receptor-ligand pairs in the PROS signaling pathway. **(G)** Circular plot showing the involvement and signal strength of each cell type in specific receptor-ligand signaling pathways. **(H)** Bubble plot of receptor-ligand pairs specifically activated in the tumor group compared to the normal group, with MP3 as the signal source. **(I)** Signal network analysis of cell types involved in the WNT signaling pathway.

Intercellular crosstalk is pivotal for disease progression. To explore cellular communication networks during disease development, we employed the CellChat platform. While the tumor group exhibited communication frequencies comparable to the normal group, signal intensities were significantly elevated (**Figure 5a**). Notably, the signaling landscape was distinctly reshaped: whereas CD56^+^ NK cells served as primary signal receivers in normal tissue, LA_TAM cells became the dominant recipients in tumors. Enhanced signal sending and receiving by MP3 and MP4 epithelial subpopulations indicate functional remodeling under tumorigenic conditions (**Figure 5b**).

Further analysis identified several additional pathways with distinct activation patterns, including chemokine-driven immune surveillance, immunosuppressive axes, and growth/angiogenesis factors. Although the TRAIL pathway (frequently subject to tumor resistance (60)). and IL-2 signaling (often co-opted in the TME (61)) showed notable activity, their overall predictive scores were comparatively lower (**Figures 5c, d**).

Among the identified circuits, the PROS1-AXL/MERTK axis emerged as the most robust interaction. MP3 epithelial cells are computationally predicted to act as primary ligand emitters, while Inflam_TAMs and LA_TAMs function as major receivers (**Figures 5e–g**). The PROS1-TAM signaling axis is of particular interest, as it is predicted to suppresses macrophage M1 polarization and antitumor immunity. Tumor-derived PROS1, frequently upregulated by IFN-γ, is predicted to activate Mer and Tyro3 receptors on macrophages, which is consistent with mechanisms that inhibit p38α phosphorylation and the subsequent expression of M1 cytokines such as IL-1 and IL-6. Rather than simply promoting M2 polarization, this interaction is predicted to actively suppress M1 activation to shape an immunosuppressive tumor microenvironment (62). Furthermore, the MIF-(CD74^+^CXCR4/CD44) network showed strong enrichment in the tumor microenvironment, suggesting a predicted role in myeloid cell recruitment and activation (**Figure 5h**). Concurrently, pDCs were found to specifically emit WNT signals that predominantly targeted MP3 (**Figure 5i**). It is important to note that these interactions are inferred from ligand-receptor expression co-occurrence; they should be interpreted as hypothesis-generating models requiring direct experimental validation.

### 3.6 MP3 subgroup-related features can predict patient survival

We evaluated molecular features linked to HCC prognosis by integrating data from TCGA-LIHC, ICGC, and OEP000321 with their associated clinical outcomes (**Supplementary Tables 2**–**4**). An intersection analysis of MP3-specific DEGs and tumor-normal epithelial cell DEGs identified 615 candidate genes for predictive modeling. We developed a robust prediction framework using the MIME framework, integrating penalized regression and ensemble learning. From an initial pool of candidate genes, 10 genes with independent prognostic significance were retained for modeling. The final TEFS model was constructed using the StepCox [both] + RSF algorithm, resulting in a parsimonious five-gene signature. This stepwise reduction from 10 to 5 variables effectively lowered model complexity while retaining key biological signals. Internal 10-fold cross-validation yielded an average C-index of 0.733. Crucially, model selection was driven by external validation performance: TEFS demonstrated consistent predictive capacity in independent cohorts (ICGC: C-index = 0.64; OEP000321: C-index = 0.65). This performance gradient, rather than indicating overfitting, reflects the rigorous external-validation-driven selection process that successfully prevented excessive fitting to training-specific noise.

**Figure 6:**
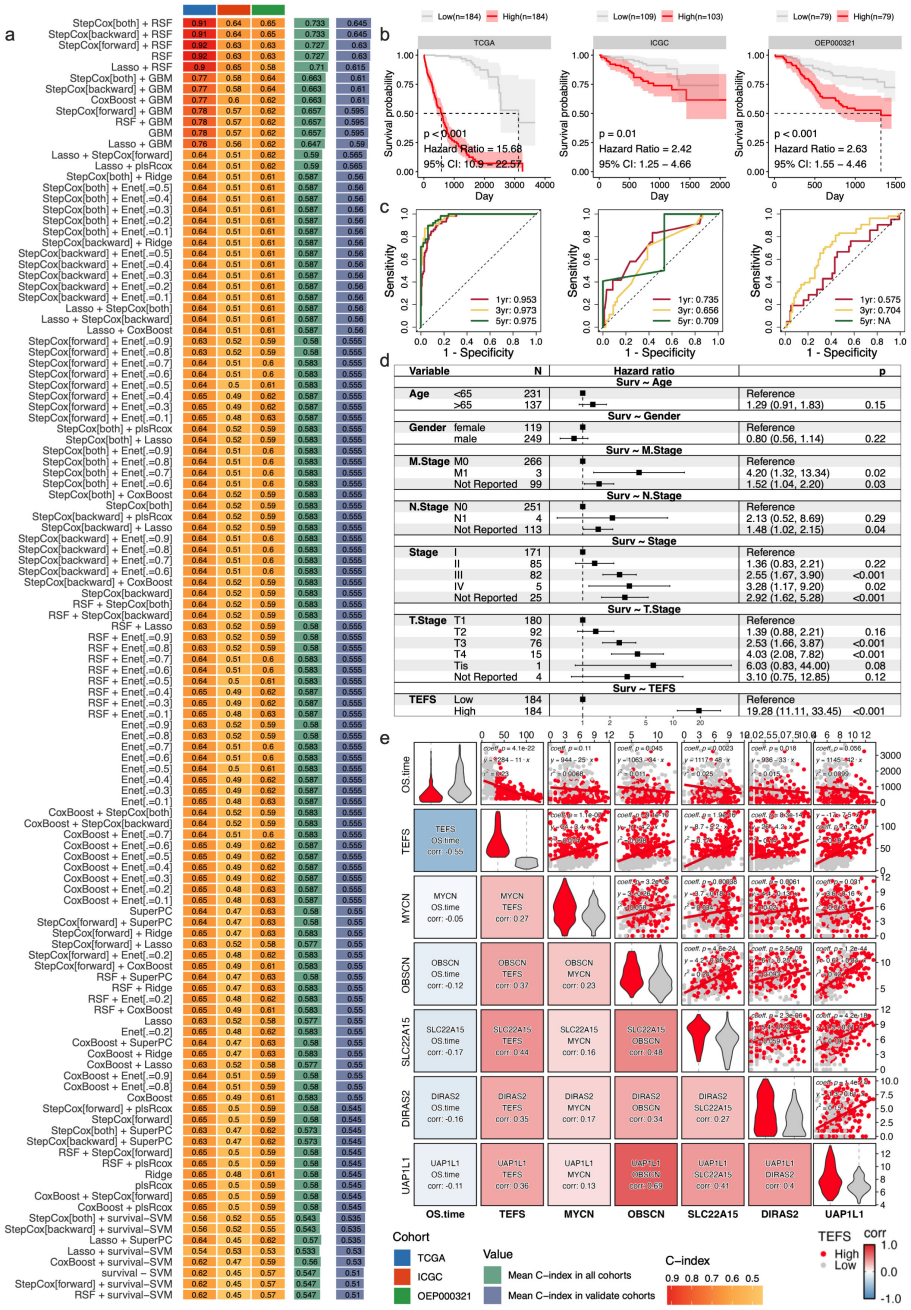
Machine learning-based prognostic model construction for HCC. **(A)** Construction and validation of the consensus feature signature TEFS using a machine learning-based ensemble approach. The C-index of all models on the training and validation sets is shown in detail. **(B)** Kaplan-Meier survival curves of the TEFS prognostic model in training and validation cohorts. **(C)** ROC curve of the TEFS prognostic model in training and validation cohorts, with 1-year, 3-year, and 5-year AUC values used to evaluate the model’s performance. **(D)** Multivariate Cox regression analysis confirms that the TEFS score is an independent prognostic factor for HCC patients. **(E)** Correlation analysis showing the relationship between modeling genes and both overall survival (OS) and TEFS scores.

The TCGA dataset served as the training set, with the ICGC and OEP000321 datasets used for validation. TEFS demonstrated strong discriminative capacity in the training cohort (C-index = 0.91), with moderate but consistent performance in the external validation sets (ICGC: 0.64; OEP000321: 0.65) (**Figure 6a**). Kaplan-Meier analysis revealed significantly longer overall survival (OS) in the low-risk group across all cohorts (p < 0.001; **Figure 6b**). Time-dependent ROC analysis showed excellent 1-, 3-, and 5-year Area Under the Curve (AUC) in the training set (0.953, 0.973, and 0.975, respectively). In the ICGC cohort, the corresponding AUCs were 0.735, 0.656, and 0.709 (**Figure 6c**). The OEP000321 cohort exhibited moderated discrimination (1-year AUC: 0.575; 3-year AUC: 0.704), with 5-year AUCs incalculable due to limited follow-up.

To assess independence, multivariate Cox regression was performed in TCGA-LIHC, adjusting for age, sex, T/N/M stage, and overall stage. TEFS remained a highly significant independent predictor of OS (Hazard Ratio (HR) = 19.28, 95% confidence interval (CI): 11.11–33.45; p < 0.001) (**Figure 6d**), providing a quantifiable, biology-informed metric for risk stratification. Gene-level correlation analysis confirmed that all constituent markers exhibited significant negative correlations with OS (**Figure 6e**).

Benchmarking against 20 previously reported HCC prognostic signatures revealed that TEFS consistently achieved the highest C-index and maintained statistical significance across all three cohorts, whereas most pathway-specific models showed inconsistent validation (**Supplementary Figures 3a, b**). TEFS also demonstrated superior or comparable 1-, 3- and 5-year AUCs across available cohorts (**Supplementary Figures 3c–e)**. This robust performance likely stems from TEFS’ cell-state-driven construction, which anchors to the MP3 malignant epithelial program to capture coupled metabolic reprogramming, immune checkpoint upregulation, and myeloid-recruiting signals. By resolving terminal malignant phenotypes rather than averaging heterogeneous bulk signals, TEFS reduces non-tumor cell noise.

### 3.7 Prognostic features of TEFS in the representation of the tumor immune microenvironment, mutation landscape, and drug sensitivity prediction

**Figure 7:**
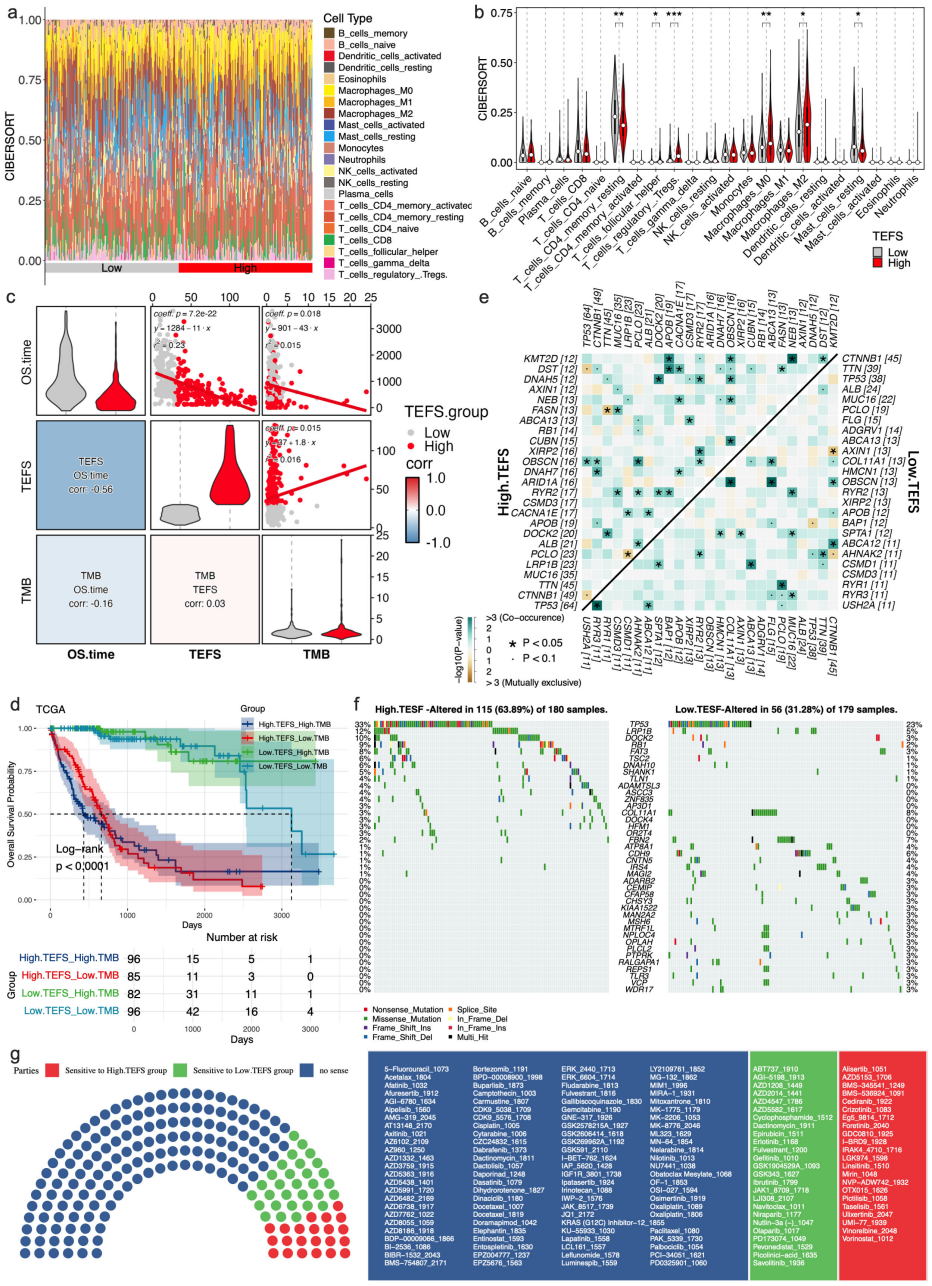
Immune infiltration microenvironment remodeling, mutational profiling, and drug sensitivity analysis based on TEFS scores. **(A)** Immune infiltration analysis of high- and low-risk groups identified by the model, using the CIBERSORT method. **(B)** Violin plots showing the infiltration levels of various immune cell types. **(C)** Correlation of tumor mutational burden score with TEFS score and OS. **(D)** Kaplan-Meier curve analysis of OS combining TMB score and TEFS score. **(E)** Heatmap showing the relationship between co-occurring and exclusive mutations in the top 25 mutated genes in the high- and low-risk groups. **(F)** Waterfall plot of the low-TEFS group showing mutations. **(G)** Drug sensitivity analyses between low- and high-TEFS groups. *, P<0.05; **, P<0.01; ***, P<0.001.

To investigate the link between TEFS and the tumor immune microenvironment, we assessed immune cell infiltration in the high- and low-risk groups using CIBERSORT. The analysis revealed a significantly elevated proportion of Treg cells and M2 macrophages in the high-risk group, alongside a marked reduction in resting CD4^+^ memory T cells (**Figures 7a, b**). This altered immune landscape suggests a more immunosuppressive microenvironment in high-risk patients, driven by an expansion of Tregs and M2 macrophages. These observations align partially with our earlier single-cell analysis.

To investigate potential links between genomic alterations and the immune landscape, we conducted comparative mutation analyses according to TEFS grouping. Tumor mutation burden (TMB) did not differ significantly between the high-risk and low-risk groups (**Figure 7c**). Stratifying tumor samples by both TMB and TEFS identified four distinct subgroups. Kaplan-Meier survival analysis revealed significant prognostic differences among these subgroups, with the most favorable clinical outcomes observed in the low-risk, high-TMB subgroup (**Figure 7d**). We further examined the co-occurrence and mutual exclusivity of mutations in the top 25 mutated genes between the risk groups. The high-risk group exhibited a greater frequency of co-occurring mutations, consistent with previous studies that reported specific genotypes are closely linked to metastatic disease and poor prognosis in prostate cancer, highlighting the critical role of genetic background in determining cancer aggressiveness and survival (63). Mutation frequencies were significantly higher in the high-risk group than in the low-risk group (63.89% vs. 31.28%), particularly for TP53 (33% vs. 23%), LRP1B (12% vs. 5%), and DOCK2 (10% vs. 3%). Several group-specific mutations were also identified, including ADAMTSL3, ASCC3, and ZNF835 exclusive to the high-risk group, and ADARB2, CEMIP, and CFAP58 unique to the low-risk group (**Figures 7e, f**).

We further predicted the half-maximal inhibitory concentrations (IC_50_) of 146 chemotherapeutic agents based on drug sensitivity analysis. The high-risk group showed greater sensitivity to 22 of these drugs, as reflected by their lower IC_50_ values. Conversely, patients in the low-risk group were sensitive to 25 different drugs (**Figure 7g**). These findings indicate that the risk score may predict differential drug sensitivity, offering a perspective for understanding tumor treatment and resistance.

### 3.8 UAP1L1 is associated with hepatocellular carcinoma progression and is computationally predicted to modulate the immune-metabolic microenvironment

**Figure 8:**
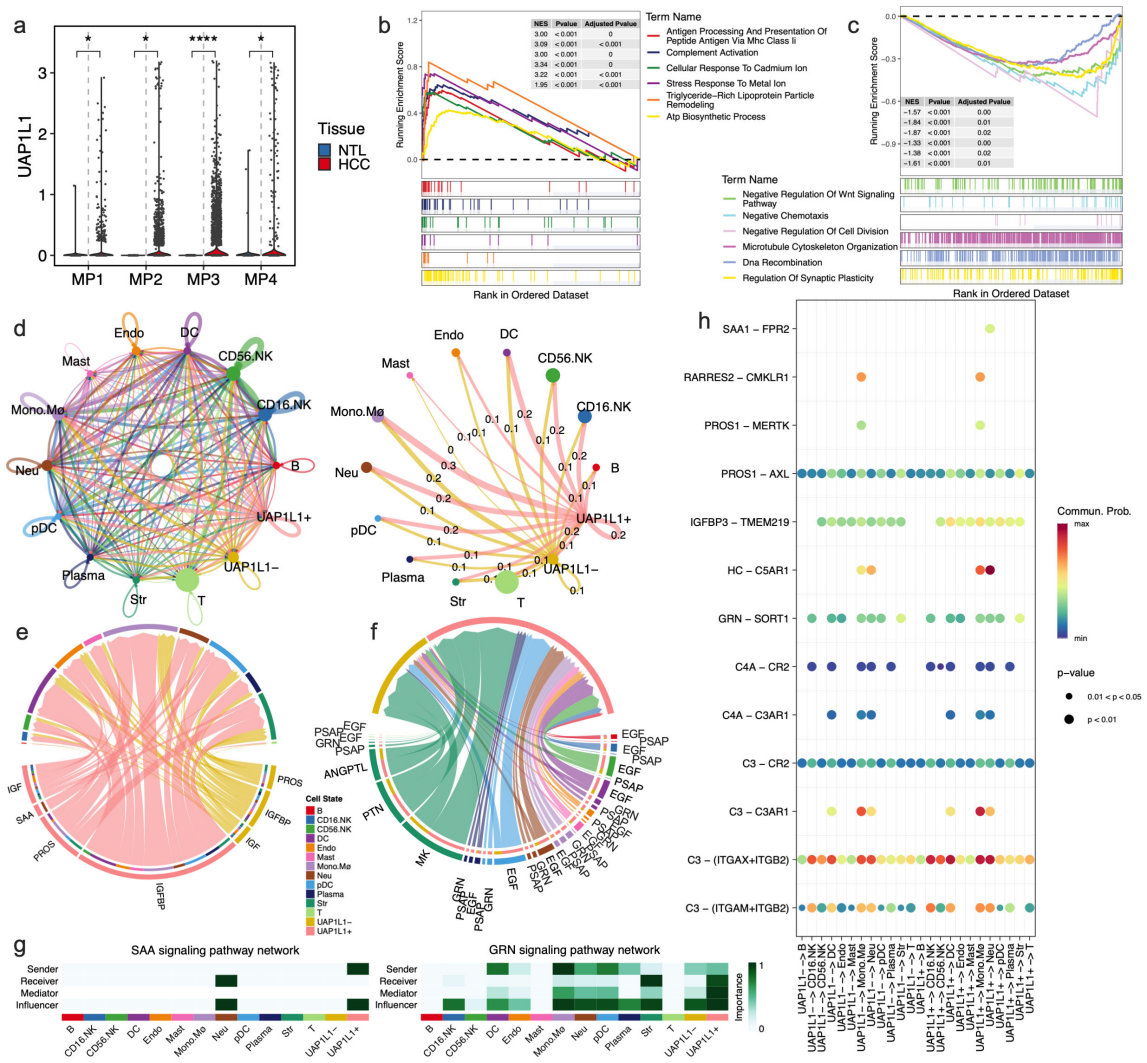
UAP1L1 expression characteristics and its functional analysis in HCC cell communication network. **(A)** Differential expression of the UAP1L1 across different programs and between normal and tumor groups. **(B)** GSEA enrichment analysis revealing representative signaling pathways associated with upregulated genes in the UAP1L1-positive group. **(C)** GSEA enrichment analysis revealing representative signaling pathways associated with upregulated genes in the UAP1L1-negative group. **(D)** Circular plot displaying the interaction network strength between UAP1L1-positive/negative epithelial cells and various immune cells. **(E)** Analysis of key signaling pathways by which UAP1L1-positive/negative populations act as ligand emitters. **(F)** Analysis of the main signaling pathways received by UAP1L1-positive/negative populations as signal receptors. **(G)** Heatmap shows the scores of each cell type involved in the SAA and GRN signaling pathways, including their roles as Senders, Receivers, Mediators, and Influencers. **(H)** Bubble plots illustrating the interaction patterns between UAP1L1-positive/negative epithelial cells and immune cells. *, P<0.05; ****, P<0.0001.

Among the five characteristic genes of TEFS, UAP1L1 has drawn particular interest. Prior studies have identified UAP1L1 as a pan-cancer oncogenic factor in several malignancies (64–67). Its role in HCC, however, remains undefined. We first examined UAP1L1 expression across epithelial subtypes, noting marked upregulation in most tumor epithelial cells, with the most pronounced elevation in the MP3 subtype (**Figure 8a**). This expression pattern prompted us to investigate its potential role in HCC progression.

To elucidate the functional context, we performed differential expression and GSEA analyses comparing UAP1L1-positive (UAP1L1^+^) and UAP1L1-negative (UAP1L1^-^) epithelial cells. UAP1L1^+^ cells exhibited significant upregulation of “immune-metabolic” pathways, including antigen presentation, complement activation, metal ion detoxification, oxidative phosphorylation, and lipoprotein remodeling(**Figure 8b**). These features are consistent with a pro-tumorigenic phenotype that may facilitate immune evasion and metabolic adaptation. Conversely, UAP1L1^-^ cells showed enrichment in pathways related to DNA repair, microtubule organization, and negative regulation of Wnt signaling (**Figure 8c**), a profile aligning with a phenotype more susceptible to immune surveillance.

We next analyzed predicted cell-cell communication networks involving UAP1L1^+^ and UAP1L1^-^ epithelial cells (**Figures 8d–g**). Two signaling axes emerged as particularly robust: the SAA pathway and the GRN signaling network. UAP1L1^+^ cells are computationally predicted to be prominent sources of SAA signals, which are consistent with reported roles in neutrophil recruitment and polarization toward an N2-like phenotype, potentially linked to NET formation and immunosuppressive features (68–70). Concurrently, UAP1L1^+^ cells are predicted to receive GRN signals, a pattern that aligns with EMT, angiogenesis, and the secretion of immunosuppressive cytokines such as IL-10 and TGF-β (71). While additional ligand-receptor pairs (e.g., IGFBP, PROS, IGF, and complement components) were detected, their interaction scores were comparatively lower (**Figure 8h**).

Collectively, these computational predictions suggest that UAP1L1^+^ epithelial cells may contribute to an immunosuppressive and pro-metastatic microenvironment through SAA-mediated neutrophil modulation and GRN-associated remodeling programs. It is important to emphasize that these findings are derived from transcriptomic co–expression and ligand-receptor prediction algorithms; they should be interpreted as a hypothesis-generating framework. Direct experimental validation, including *in vitro* co-culture and *in vivo* models, is required to confirm the causal roles of these predicted interactions in HCC progression.

### 3.9 UAP1L1 knockdown suppresses HCC cell viability and migration *in vitro*

**Figure 9:**
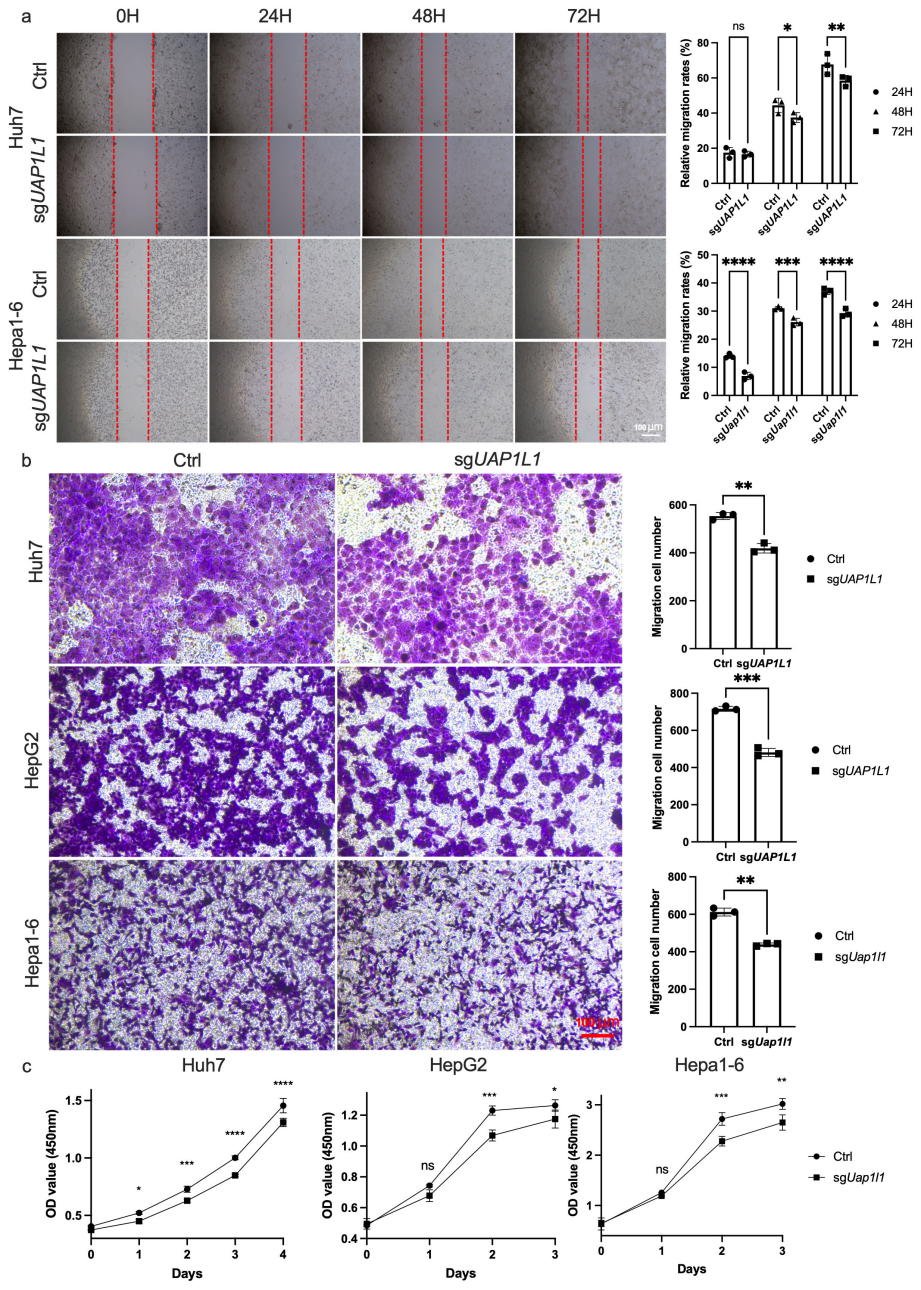
UAP1L1 promotes HCC cells proliferation and invasion. **(A)** Wound healing assays revealed a decrease in the migration ability of Huh7 and Hepa1–6 cells in the sg*UAP1L1* group compared to the Ctrl group. Quantification of Wound healing assays results showing a decrease in wound closure percentage in the sg*UAP1L1* group compared to the Ctrl group. Scale bar: 100 μm. **(B)** Transwell assay demonstrated a decrease in the migration ability of Huh7, HepG2 and Hepa1–6 cells in the sg*UAP1L1* group compared to the Ctrl group. Quantification of Transwell assay results showing a decrease in the number of invading cells in the sg*UAP1L1* group compared to the Ctrl group. Scale bar: 100 μm. **(C)** CCK-8 assay further confirmed the inhibitory effect of *UAP1L1* silencing on Huh7, HepG2 and Hepa1–6 cells proliferation. *, P<0.05; **, P<0.01; ***, P<0.001; ****, P<0.0001.

To investigate the function of *UAP1L1* in HCC, we knocked out its expression in Huh-7, HepG2, and Hepa1–6 cells using CRISPR/Cas9-mediated knockout. RT-qPCR experiments confirmed that *UAP1L1*/*Uap1l1* was stably knocked out in the constructed cell lines. (**Supplementary Figure 4a**). Wound healing assays first demonstrated a pronounced decrease in cell migration following *UAP1L1* depletion (**Figure 9a**). This result was further supported by Transwell assays, which confirmed that *UAP1L1* knockout inhibits cell migration (**Figure 9b**). Finally, CCK-8 assay revealed that *UAP1L1* knockout significantly reduced cell proliferation (**Figure 9c**). We also examined immunohistochemical (IHC) staining data for UAP1L1 from the Human Protein Atlas (HPA) database, and the results confirmed that UAP1L1 protein levels are downregulated in HCC. (**Supplementary Figure 5**).

## 4 Discussion

HCC is a globally prevalent malignancy characterized by significant molecular heterogeneity and a complex tumor microenvironment (1, 2). Although traditional treatments including surgery, chemotherapy, and targeted therapy have advanced, the prognosis for patients with advanced disease remains poor (6, 7). Here, we analyzed 65 samples from four public scRNA-seq datasets, revealing the cellular diversity of stromal and immune components and identifying a key immunotolerant epithelial state (MP3) in the dynamic progression of HCC. Rather than introducing a completely new paradigm, this work positions itself as a single-cell-resolution refinement and integration of existing concepts in metabolic reprogramming, immunosuppression, and myeloid remodeling. While numerous bulk-derived prognostic signatures have been developed for HCC [e.g., focusing on isolated pathways such as ferroptosis (72), necroptosis (73, 74), or lncRNA networks (73–75)], they primarily rely on tissue-level averages and struggle to resolve the heterogeneity of malignant epithelial states. In contrast, TEFS is directly anchored to the MP3 terminal epithelial program, which biologically encodes coupled metabolic reprogramming, immune checkpoint upregulation, and myeloid-recruiting signals. Rather than introducing entirely novel targets, TEFS’ novelty lies in unifying previously fragmented metabolic and immune indicators under a cell-state-driven framework. This approach mitigates the averaging effects of bulk sequencing and likely explains its superior internal performance (C-index 0.733) and more consistent external validation across independent cohorts compared to pathway-specific models. By capturing a coherent terminal malignant phenotype, TEFS serves as a compressed molecular readout that refines how existing concepts of metabolic dysregulation, immunosuppression, and myeloid remodeling are orchestrated by specific epithelial trajectories.

Our cellular communication analysis underscores the central role of the PROS1-AXL/MERTK axis and the MIF network in HCC progression (62, 76). The predicted crosstalk between MP3 epithelial cells and LA_TAMs via PROS1 aligns with emerging evidence of ligand-driven macrophage tolerance (77), while MIF-mediated signaling may facilitate myeloid recruitment and profoundly reprogram the local metabolic landscape. Recent studies highlight MIF as a pivotal regulator bridging inflammation and tumor metabolic plasticity, where it orchestrates glucose uptake, glycolytic flux, and lipid/amino acid utilization through receptor networks such as CD74 and CXCRs (76). This computationally inferred metabolic-immune crosstalk likely establishes a nutrient-rich, immunosuppressive niche that supports LA_TAM survival and function (78). We emphasize that these findings are computationally inferred and should be viewed as a hypothesis-generating model. Future studies employing *in vivo* co-culture systems, pathway inhibition, or spatial transcriptomics will be essential to validate whether UAP1L1^+^ epithelial cells and LA_TAMs functionally interact through these predicted axes to drive immunosuppression and metabolic remodeling.

This study integrated multiple single-cell datasets to comprehensively characterize the functional properties of tumor cells during HCC progression and their influence on the immune microenvironment. It specifically underscores the central role of MP3 stage epithelial cells in driving tumor advancement. These cells are associated with progression features that may involve diverse mechanisms, such as regulating proliferation, modulating apoptosis, and remodeling the microenvironment.

We identified LA_TAMs as a critical terminal state of myeloid cells, which were significantly enriched tumor microenvironments. This distinct enrichment suggests that LA_TAMs may play a specialized role in HCC progression, potentially shaping an immunosuppressive landscape by inhibiting effector T cells and NK cells, thereby facilitating tumor immune evasion (40, 51, 52). These findings elucidate immune escape mechanisms associated with this tumor-associated macrophage subset and suggest that targeting the UAP1L1/MIF-LA_TAM metabolic-immune axis may offer new therapeutic avenues. However, the clinical translation of these insights will require prospective validation to distinguish context-dependent immune functions from tumor-promoting activities.

The TEFS prognostic model, anchored to the MP3 terminal epithelial program, demonstrated robust predictive accuracy across internal and external cohorts. To ensure generalizability, we employed 10-fold cross-validation combined with the RSF ensemble algorithm and external validation-driven model selection. The low dimensionality of the final 5-gene model (EPV > 20) relative to the training cohort (n = 368) inherently limits over-parameterization. Furthermore, the RSF approach leverages bootstrap sampling and random feature selection to reduce variance, while the MIME framework’s strategy of prioritizing stable performance across independent validation cohorts (total n = 370) over peak training metrics effectively mitigates overfitting—a common pitfall in single-cohort bulk signatures. Multivariate Cox analysis, adjusted for age, sex, TNM staging, and overall clinical stage, confirmed TEFS as an independent prognostic factor (HR = 19.28, p < 0.001), outperforming 20 established HCC signatures in cross-cohort consistency.

The superior and more stable performance of TEFS can be biologically contextualized. Unlike bulk-derived models that average tissue-level signals and restrict features to isolated pathways (e.g., ferroptosis or lncRNA networks), TEFS captures a coherent terminal malignant phenotype driven by the MP3 program. This cell-state framework unifies fragmented metabolic and immune dysregulation into a compressed molecular readout, reflecting coupled metabolic reprogramming, immune checkpoint upregulation, and myeloid recruitment. UAP1L1 emerged as a central hub gene, computationally linked to an immunosuppressive ligand-receptor network (e.g., SAA (68–70) and GRN (71) signaling) and validated *in vitro* by attenuated proliferation and migration upon knockdown. Furthermore, cellular communication analysis highlights the PROS1-AXL/MERTK axis and MIF-mediated signaling as key drivers of epithelial-LA_TAM crosstalk, aligning with emerging evidence of ligand-driven macrophage tolerance and metabolic reprogramming. While these insights are computationally inferred and require spatial or *in vivo* validation, they provide a mechanistic rationale for TEFS’ discriminative capacity –and clinical interpretability.

## 5 Limitations and future directions

Despite these promising findings, several limitations must be acknowledged. First, our identification of MP3-associated communication networks relies heavily on computational inference from public transcriptomic data. The absence of spatial transcriptomics or immune co-culture experiments means the proposed UAP1L1-centered circuit remains hypothesis-generating.

Second, the TEFS prognostic model exhibits strong internal performance but modest external generalizability. This performance decline highlights cohort-specific biases and ethnic variations, reinforcing that TEFS should currently be viewed as an exploratory, biology-informed signature rather than a ready-to-use clinical tool. Prospective validation in diverse, multi-center cohorts and head-to-head comparison with established clinical scoring systems (e.g., BCLC, Child-Pugh, TNM) are essential before clinical translation.

Third, reliance on heterogeneous public datasets and batch-integration methods may introduce residual technical bias, and the lack of protein-level validation (e.g., CPTAC or WB) limits definitive translational claims. Future studies integrating spatial multi-omics, experimental perturbation, and clinical cohorts will be crucial to validate these findings and refine TEFS for routine HCC risk stratification.

## Data Availability

Publicly available datasets were analyzed in this study. This data can be found here: The TCGA-LIHC dataset served as the training cohort and was obtained from the UCSC platform. For validation, cohort OEP000321, comprising 158 HCC patients with transcriptomic sequencing and clinical data, was sourced from the NODE database. A further 212 HCC cases with corresponding transcriptomic sequencing and clinical data were acquired from the ICGC Data Portal. Single-cell RNA sequencing (scRNA-seq) data for 39 HCC patients and 26 normal controls were retrieved from the GEO database under accession numbers GSE149614, GSE189903, GSE202642, and GSE245906. Following quality control, 265,125 high-quality single cells from this combined dataset were retained for subsequent analysis.
