# Supplementary material for: Uncovering cellular perturbations and key mediator communications in liver cancer using single-cell RNA sequencing
Source: Front Immunol. 2026 Jul 1;17:1868090. doi: 10.3389/fimmu.2026.1868090 (PMC13369601; doi:10.3389/fimmu.2026.1868090)
Supplement: Supplementary file 1 [file DataSheet1.pdf]

Supplementary Figure 1

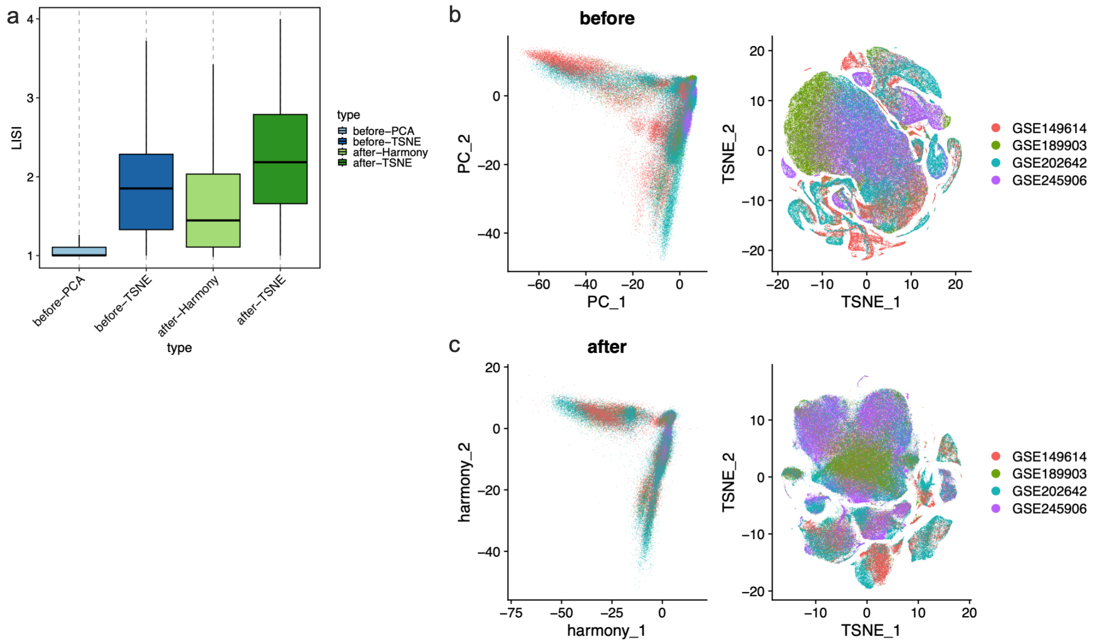

Figure S1: Batch Effect Correction in Single-Cell RNA-seq Data.

(A) The box plots illustrate the Local Inverse Simpson's Index values of the dataset before and after batch effect removal. (**B-C)** The t-SNE plots depict the distribution of the dataset before and after batch effect correction.

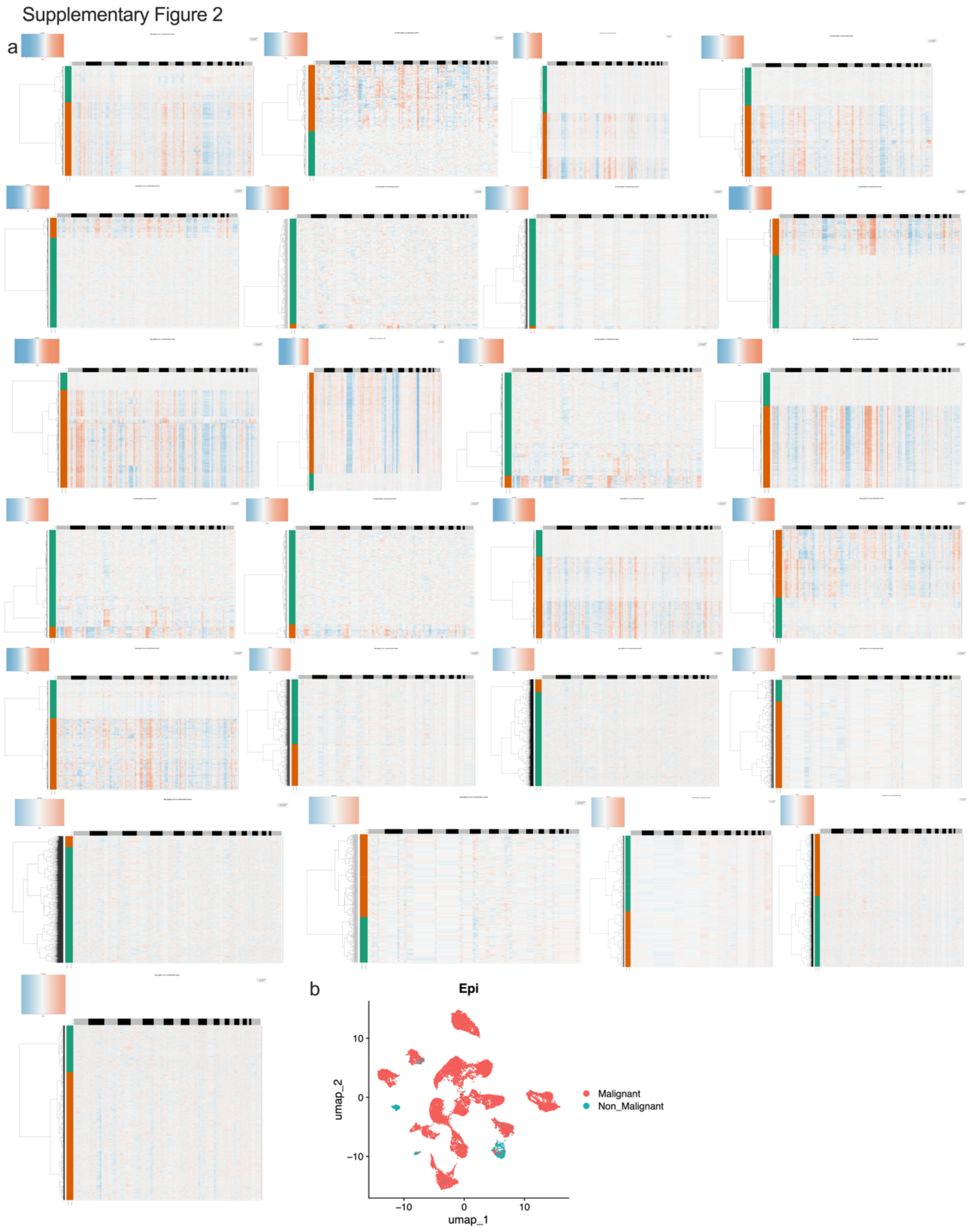

Figure S2: CopyKat analysis predicting copy number variations in each epithelial cell subtype using T cells and NK cells as references.
(A) CopyKat analysis predicting copy number variations in each epithelial cell subtype using T cells, and NK cells as references. b The UMAP plot illustrates the distribution of normal epithelial cells and malignant epithelial cells in the CopyKat analysis.

Supplementary Figure 3

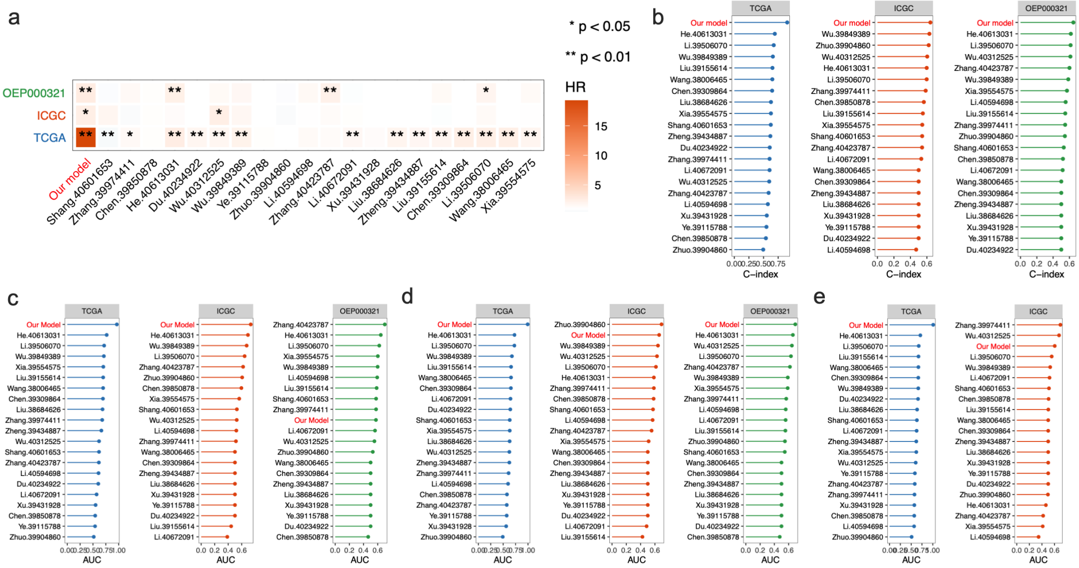

Figure S3: Comparative performance evaluation of TEFS against 20 established prognostic models across three independent cohorts.

(A) The heatmap displays the prognostic performance of TEFS alongside 20 established prognostic models across the TCGA, ICGC, and OPE000321 datasets. **(B)** The lollipop plot presents the C-index values for TEFS and 20 established prognostic models across the TCGA, ICGC, and OPE000321 datasets. (**C-E)** The lollipop plot presents the 1-, 3-, and 5-year AUC values for TEFS and 20 established prognostic models across the TCGA, ICGC, and OPE000321 datasets.

Supplementary Figure 4

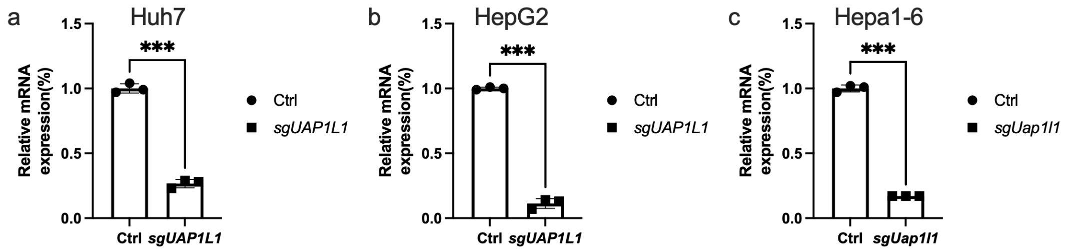

Figure S4: Knockdown efficiency of *UAP1L1*/*Uap1l1* gene detected by RT-qPCR
(A) Knockdown efficiency of *UAP1L1* gene detected by RTqPCR in Huh7 cell line. **(B)** Knockdown efficiency of *UAP1L1* gene detected by RTqPCR in HepG2 cell line. **(C)** Knockdown efficiency of *Uap1l1* gene detected by RTqPCR in Hepa1-6 cell line.

Supplementary Figure 5

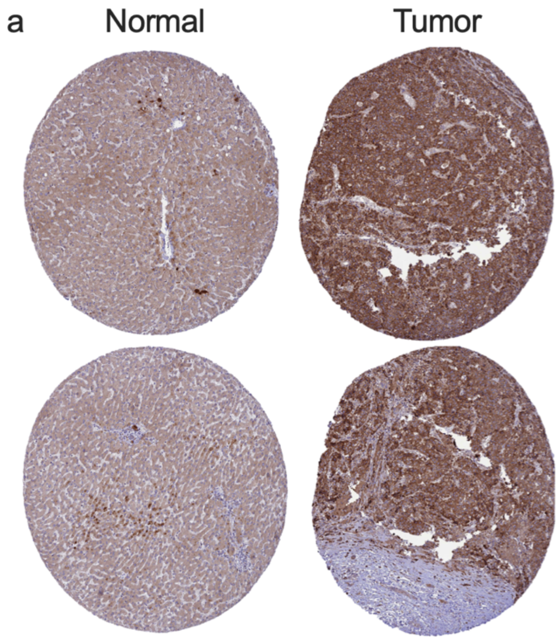

Figure S5: Immunohistochemical analysis of *UAP1L1* expression.
(A) Immunohistochemistry staining of *UAP1L1* protein expression from the HPA database.
